# Static magnetic fields in regenerative medicine

**DOI:** 10.1063/5.0191803

**Published:** 2024-03-13

**Authors:** Wenjing Xie, Chao Song, Ruowen Guo, Xin Zhang

**Affiliations:** 1 Institutes of Physical Science and Information Technology, Anhui University, Hefei, Anhui 230039, China; 2 High Magnetic Field Laboratory, Key Laboratory of High Magnetic Field and Ion Beam Physical Biology, HFIPS, Chinese Academy of Sciences, Hefei, Anhui 230031, China

## Abstract

All organisms on Earth live in the weak but ubiquitous geomagnetic field. Human beings are also exposed to magnetic fields generated by multiple sources, ranging from permanent magnets to magnetic resonance imaging (MRI) in hospitals. It has been shown that different magnetic fields can generate various effects on different tissues and cells. Among them, stem cells appear to be one of the most sensitive cell types to magnetic fields, which are the fundamental units of regenerative therapies. In this review, we focus on the bioeffects of static magnetic fields (SMFs), which are related to regenerative medicine. Most reports in the literature focus on the influence of SMF on bone regeneration, wound healing, and stem cell production. Multiple aspects of the cellular events, including gene expression, cell signaling pathways, reactive oxygen species, inflammation, and cytoskeleton, have been shown to be affected by SMFs. Although no consensus yet, current evidence indicates that moderate and high SMFs could serve as a promising physical tool to promote bone regeneration, wound healing, neural differentiation, and dental regeneration. All *in vivo* studies of SMFs on bone regeneration and wound healing have shown beneficial effects, which unravel the great potential of SMFs in these aspects. More mechanistic studies, magnetic field parameter optimization, and clinical investigations on human bodies will be imperative for the successful clinical applications of SMFs in regenerative medicine.

## I. INTRODUCTION

Regenerative medicine is an emerging multidisciplinary science that aims to develop methods to regrow, repair, or replace damaged/diseased cells, organs, or tissues^1^ with the goal of restoring our physiology to its original condition and showing promising results across multiple systems. A growing body of data indicates that magnetic field has the potential to be used in regenerative medicine. Depending on whether magnetic fields change over time, they can be divided into time-varying magnetic fields and static magnetic fields (SMFs). The focus of this article is SMFs, whose intensity and direction are constant. As the fundamental units of regenerative medicine, stem cells are undifferentiated cells with clonal potential, capable of self-renewal and differentiation,^3^ and are mainly responsible for the regeneration and development of organs and tissues.^2,4^ It is interesting that stem cell is one of the most sensitive cell types to SMFs. In fact, there have been several studies demonstrating the effects of SMFs on dental pulp stem cells (DPSCs), bone marrow mesenchymal stem cells (BMSCs), and human adipose-derived stem cells (ASCs).^5–7^ In addition, SMFs have been shown to promote tissue repair, expedite wound healing, and to maintain bone health.^8^ The purpose of this review is to summarize the research progress of SMFs on regenerative medicine related aspects, including animal and cellular studies about bone regeneration, wound healing, nerve, dental pulp, and muscle. It is obvious that both positive and negative results are present in the literature, and we try to summarize and analyze them to find some consensus, including SMF parameters and biological systems with beneficial effects, which could provide a foundation for clinical exploration, as well as future systematic and mechanistic studies.

## II. BONE REGENERATION

Bone regeneration has always been a hot topic in magnetic fields and regenerative medicine. Studies have shown the positive effects of pulsed electromagnetic fields (PEMFs) in bone regeneration, which generate weak electrical currents in bone through external coils on the cast or skin. Food and Drug Administration (FDA) has approved it as an aid in osteoporosis and osteoarthritis treatment. For this aspect, there are multiple related reviews where people can find more information.^9–11^ For SMFs, although they have not been FDA-approved, there are also quite a few reports related to its potential application in bone regeneration (Table I), which show that SMFs have multiple beneficial effects on the bone systems both *in vitro* and *in vivo*.

**TABLE I.** Effects of SMFs on bone regeneration. Bone marrow mesenchymal stem cells (BMSCs); periodontal ligament stem cells (PDLSCs); Wharton's jelly mesenchymal stem cells (WJMSCs); mandibular condylar chondrocytes (MCCs); and N/A, not available.

Objects			Magnetic flux density	Magnetic field direction	Exposure time	Effects on cell proliferation and differentiation	Effects	References
*In vitro*	Humans	BMSCs	3, 15, and 50 mT	Upward	21 days	Promotion	Promoted proliferation and osteoblast differentiation of human BMSCs	18
			50, 100, and 150 mT	Perpendicular	12 h/day, 14 days		Promoted osteogenic differentiation of cells	19
			100 mT	Upward	28 days		Enhanced cell viability, DNA synthesis, and led to early transformation of the osteogenic lineage supported by Runx2 and ALP expression	20
				Perpendicular	12 h/day, 1, 4, 7, and 14 days		Enhanced cell viability, proliferation and adhesion, improved cell morphology, and promoted osteogenic differentiation, possibly due to upregulation of the BMP-Smads signaling pathway	33
			100, 200, 400, and 600 mT		7, 14, and 21 days		Induction of chondrogenesis in BMSCs via TGF-β-dependent pathway	21
		PDLSCs	320 mT	N/A	14 days		Activation of the phosphorylated AKT pathway to enhance proliferation and osteogenic differentiation	24
		WJMSCs	400 mT				Increased matrix vesicle secretion and mineralization, enhanced osteogenic differentiation, but no effect on cell viability and growth	34
		Chondrocytes	3 T		1 and 96 h	Inhibition	Suppressed cell growth and induced apoptosis through p53, p21, p27, and Bax protein expression	25
	Rats	Calvaria cell	160 mT	N/A	20 days	Promotion	The values of the total area and the number and average size of bone nodules showed high levels, the calcium content in the matrix and alkaline phosphatase and OCN also showed a significant increase	14
		Mandibular BMSCs/MCCs	280 mT	Vertical	14 days		Improved chondrogenesis and proliferation of Mandibular BMSC in the co-culture system	22
		MCCs		N/A			Enhanced differentiation through FLRT/BMP signaling	23
	Mice	Osteoblast MC3T3-E1	500 nT, 200 mT, and 16 T	Upward and downward	10 days	Promotion/inhibition	16 T SMF increased osteoblast differentiation, 500 nT and 200 mT SMFs decreased osteoblast differentiation	12
		Raw264.7		N/A			500 nT and 200 mT SMFs promoted the differentiation, formation, and resorption of osteoclast Raw264.7, whereas a 16 T SMF acted as an inhibitor	13
			1–2 T		4 days	Inhibition	Osteoclast formation in the presence of Ferumoxytol was reduced	15
		Osteoblast MC3T3-E1	8 T	Parallel	14 days	Promotion	Stimulated bone formation to grow in the direction of the magnetic field	16
					60 h			17
*In vivo*	Rats	Whole animal	4 mT	⋯	16 weeks		Prevented bone architectural deterioration and strength reduction	30
			30–200 mT	Parallel	12 weeks		Increased bone density and bone area	31
			100 mT	N/A	12 h/day, 6 and 12 weeks		Enhanced bone regeneration and osseointegration	19
			180 mT		6 weeks		Significantly increased bone mineral density	32
					12 weeks		Prevented the decrease in bone mineral density	26
	Mice		200 mT	Alternating magnetic poles	2 weeks		Stimulated chondrogenesis, enhanced the cellular matrix of cartilage and increased endogenous stem cell migration	27
			200–400 mT	Downward	4 weeks		Increased of bone resorption in reloaded mice	28
			2–4 T	N/A	28 days		Promoted bone formation, increased osteoblasts, and reduced the number of osteoclasts	29

### A. Bone regeneration *in vitro*

Osteoblasts and osteoclasts are cells that work together to form new bones and break down old/damaged bones. Several *in vitro* experiments on osteoblasts (build new bones) and osteoclasts (dissolve old/damaged bones) have shown that SMF affects them in a magnetic field intensity and cell-type dependent manner. For example, in 2014, Shang's team found that both 500 nT hypomagnetic field and 200 mT moderate SMF decreased osteoblast differentiation, while 16 T high SMF increased osteoblast differentiation.^12^ Moreover, it is interesting that they observed opposite effects of these SMFs on osteoclasts: the 500 nT and 200 mT SMF increased osteoclast differentiation, but the 16 T SMF decreased osteoclast differentiation.^13^ These demonstrate that the effect of SMFs on bone regeneration is related to both magnetic field strength and cell type and indicate the bone promotion effects of high SMFs. Moreover, Yamamoto *et al.* treated cultured rat cranial osteoblasts with 160 mT SMF for 20 days and found increased values of the total area, number, and mean size of bone nodules.^14^ Furthermore, Zhang *et al.* found that after placing Raw264.7 cells in 1–2 T SMFs for 4 days, the osteoclast formation in the presence of Ferumoxytol was reduced.^15^ Kotani *et al.* also found that 8 T high SMF can align both mouse osteoblastic MC3T3-E1 cells and the orientation of bone formation.^16,17^

Other than osteoblasts and osteoclasts, there are also studies investigating the effects of SMFs on bone regeneration by acting on bone marrow and umbilical cord-derived mesenchymal stem cells (MSCs). For example, in 2015, Kim *et al.* exposed human BMSCs to 3, 15, or 50 mT SMFs and found that these moderate intensity SMFs promoted the proliferation and osteogenic differentiation of human BMSCs, especially at 15 mT.^18^ In 2019, He *et al.* examined the osteogenic effect of 50, 100, and 150 mT SMFs on human BMSCs in three-dimensional-printed (3DP) scaffolds and found that the SMFs promoted osteogenic differentiation of hBMSCs.^19^ Moreover, there are multiple studies reported that 200–400 mT SMFs can prevent BMSC lipogenic differentiation, increase osteogenic differentiation,^20^ stimulate chondrogenic differentiation,^21^ enhance mandibular BMSCs chondrogenesis and proliferation,^22^ accelerate mandibular condylar chondrocytes (MCCs) osteogenesis,^23^ and promote the proliferation, migration, and osteogenic differentiation of periodontal ligament stem cells (PDLSCs).^24^ SMFs can also significantly attenuate dexamethasone or all-trans retinoic acid-induced bone loss in mice.^20^

Most *in vitro* studies in the literature showed beneficial effects of SMFs on bone regeneration, with only a few exemptions. For example, Shang's team found that both 500 nT hypomagnetic field and 200 mT moderate SMF decreased osteoblast differentiation^12^ and increased osteoclast differentiation;^13^ Hsieh *et al.* found that 3 T SMF inhibited the growth of human chondrocytes *in vitro* and affected the recovery of damaged knee cartilage in pig models.^25^ However, it is interesting that as far as we know, all animal studies in the literature demonstrated beneficial effects on bone regeneration, which will be introduced below.

### B. Bone regeneration *in vivo*

Several rodent studies have been conducted to determine the effectiveness of SMFs on bone growth, which indicate that SMFs promote not only bone healing process but also bone formation *in vivo*.^8^ For example, He *et al.* used 100 mT SMF to treat a rat model of bone defect for 6 and 12 weeks and found that SMF promoted bone regeneration and osseointegration.^19^ Tapered rod-shape magnets of 180 mT were implanted transcortically into the middle diaphysis of rat femurs for 12 weeks, which prevented the loss of bone mineral density (BMD) caused by the implantation operation.^26^ Sun *et al.* treated osteoarthritis (OA) mice with 200 mT SMF for 2 weeks, which stimulated chondrogenesis, enhanced the cellular matrix of cartilage, and attenuated the pathological progression of cartilage destruction in OA mice.^27^ In 2021, the Shang's group found that 4 weeks of SMFs treatment at 200–400 mT facilitated the recovery of unloading-induced bone loss by inhibiting bone resorption in reloaded mice.^28^ In the same year, they also reported that mice exposed to 2–4 T SMFs for 28 days have improved bone microstructure and mechanical properties.^29^

There are also a few studies reported positive effects of SMFs on diabetic bone formation and postmenopausal-induced osteoporosis. For example, Zhang *et al.* found that 16 weeks of 4 mT SMF treatment can stimulate the bone formation in streptozotocin (STZ)-treated diabetic rats, prevent bone architectural deterioration and strength reduction.^30^ Taniguchi *et al.* found that 30–200 mT SMFs treatment for 12 weeks can increase the bone density and bone area of ovariectomized (OVX) rats.^31^ Xu *et al.* implanted a 180 mT small disk magnet on the right side of the lumbar spine of OVX rats and found that the bone density of the lumbar vertebrae near the magnet was significantly increased after 6 weeks.^32^

### C. Effects of SMFs combined with magnetic nanoparticles on bone regeneration

Some investigations have also been conducted to explore how the combination of SMFs with other therapies affects bone regeneration, and the focus here is on the combination of SMFs with magnetic nanoparticles (MNPs) (Table II). Combining 15 mT SMF and MNPs-added polycaprolactone (PCL) nanocomposite scaffolds was found to promote osteoblast differentiation and bone formation both *in vitro* and *in vivo*.^35^ Filippi *et al.* also observed that 50 mT SMF treatment for 21 days stimulated the osteogenic and angiogenic potential of constructs composed of MNPs doped into polyethylene glycol (PEG)-based hydrogels containing human adipose tissue stromal vascular fraction (SVF) cells.^36^ In addition, Boda *et al.* found that 100 mT SMF induced proliferation arrest of human mesenchymal stem cells (MSCs) cultured on the magnetic composite hydroxyapatite (HA)-magnetite (Fe_3_O_4_), leading to early cell differentiation and osteogenic formation.^37^ Also, Wu *et al.* cultured BMSCs with 100 mT SMF and Fe_3_O_4_ nanoparticles for 24 h and found that low doses of Fe_3_O_4_ nanoparticles combined with SMF could enhance osteogenesis and angiogenesis.^38^ While most studies showed positive effects, Yamaguchi-Sekino *et al.* reported that exposure to 7 T SMF for 3 h/day for 6 days had no effect on the expression of genes and proteins of osteogenic markers of MSCs in tricalcium phosphate (TCP)/MSC constructs.^39^

**TABLE II.** Effects of SMFs combined with MNPs on bone regeneration. Stromal vascular fraction (SVF); bone marrow mesenchymal stem cells (BMSCs); mesenchymal stem cells (MSCs); polycaprolactone (PCL); polyethylene glycol (PEG); hydroxyapatite (HA); tricalcium phosphate (TCP); and N/A, not available.

Cell models	Nanoparticles	Magnetic flux density	Magnetic field direction	Exposure time	Effects	References
Osteoblasts	MNP-added PCL nanocomposite scaffolds	15 mT	Upward	5 and 10 days	Promoted osteoblast differentiation and bone formation	35
Human SVF cells	Addition of MNPs to PEG	50 mT		21 days	Stimulated the osteogenic and angiogenic potential of engineered bone tissue grafts	36
Human BMSCs	Fe_3_O_4_ nanoparticles	100 mT	Parallel	24 h	Triggered exosomes to exert enhanced osteogenesis and angiogenesis	38
Human MSCs	HA-magnetite (Fe_3_O_4_)			30 min every other day, 28 days,	Led to early cell differentiation and promoting their osteogenic formation	37
Rat MSCs	TCP	7 T	N/A	3 h/day, 6 days	No reproducible effects on gene expression, protein expression or histology of TCP/P-1 and P-2 MSCs	39

## III. WOUND HEALING

There are also multiple studies indicating the promoting effects of SMFs on wound healing (Table III). Since impaired wound healing is very common in diabetic patients, quite a few studies have performed their wound healing experiments in diabetic rodent models. For example, Jing *et al.* demonstrated the benefits of 180 mT gradient SMF on wound healing in STZ-induced diabetic rats.^40^ Zhao *et al.* found that SMF promoted wound healing after treating diabetic rats with 230 mT SMF for 21 days.^41^ Shang *et al.* found that 600 mT SMF treatment for 14 days significantly accelerated wound closure in diabetic mice.^42^

**TABLE III.** Promotional effects of SMFs on wound healing. Bone marrow mesenchymal stem cells (BMSCs); human umbilical vein endothelial cells (HUVECs); and N/A, not available.

	Objects	Magnetic flux density	Magnetic field direction	Exposure time	Effects	References
*In vitro*	Human BMSCs	100 mT	Upward	24 h	Combined MNPs enhanced wound healing by improving angiogenesis and fibroblast function	45
	HUVECs	N/A	N/A	7 days	In combination with magnetically responsive hydrogels markedly promoted cell proliferation	46
	NIH 3T3					
*In vivo*	Humans			1 month	The patient's abdominal wound that existed for one year healed completely after one month of applying magnet therapy	44
	Rats	2.3 mT		15.3 ± 2.8 days	Accelerated wound healing	43
		80 mT		14 days	Combined magnetic responsive multifunctional hydrogel accelerated wound healing	46
		160 mT	Upward and downward	15 days	Accelerated the speed of tissue repair	47
		180 mT	Upward	19 days	Speeded up diabetic wound repair	40
		230 mT	Downward	21 days	Promoted the wound healing	41
	Mice	∼15 mT	Upward and downward	3 weeks	Increased the wound area closure rate	48
		600 mT	Alternating magnetic poles	3, 7, and 14 days	Promoted wound healing, facilitated resolution of inflammation	42

The ability of SMFs to promote wound healing has also been demonstrated in some other experiments in non-diabetic animal models. For example, the 2.3 mT magnets were placed on standardized wounds on the backs of rats, which accelerated the rate of wound healing.^43^ It is interesting that a case study involving a 51-year-old paraplegic woman with an abdominal wound that had been present for one year showed that the wound was completely healed after one month of applying a magnet to a conventional wound dressing,^44^ although no further detail about the magnet parameters was provided.

There are also experiments investigated the combined effects of SMFs with MNPs on wound healing. For example, BMSCs co-cultured with 100 mT SMF and MNPs Fe_3_O_4_ can enhance the wound healing of rats with dorsal wounds by improving angiogenesis and fibroblast function.^45^ Wang *et al.* found that 80 mT SMF combined with magneto-deformable cobalt ferrite nanoparticles/polyvinyl alcohol matrix can also accelerate rats wound healing.^46^

## IV. NERVE CELLS

The nervous system, including the brain, spinal cord, and neurons, is an important target of magnetic fields. It has been shown that SMF exposure has a strong modulatory impact on neural tissues (Table IV). For example, Ben Yakir-Blumkin *et al.* found that continuous exposure of young adult rats to low-intensity SMFs of <10 mT for 3 weeks significantly enhanced the generation of new doublecortin-expressing cells in the sub-ventricular zone (SVZ) and neocortex.^49^ Exposure of rat olfactory ensheathing cells (OECs) to 30, 50, and 70 mT SMFs led to filamentous pseudopod formation, cell morphology changes and cell migration promotion.^50^ Nakamichi *et al.* discovered that 100 mT SMF considerably reduced cell proliferation of neural progenitor cells (NPCs) and promoted their differentiation into neurons.^51^ Pacini *et al.* treated normal human neuronal cells (FNC-B4) with SMF generated by 200 mT magnetic resonance tomography for 120 min, and found that SMF caused significant FNC-B4 morphology changes and promoted neural differentiation.^52^ Prasad *et al.* stimulated human oligodendrocyte precursor cells (OPCs) with 300 mT SMF for 2 weeks (2 h/day) and observed enhanced myelin formation in oligodendrocytes, increased cell differentiation and myelination potential.^53^ Ho *et al.* discovered that 500 mT SMF increased the production of neurospheres and the proliferative activity of mouse NPCs.^54^ Eguchi *et al.* found that 8 T SMF treatment for 60 h induced parallel orientation of the Schwann cells along the SMF direction. In contrast, due to the strong diamagnetic anisotropy of collagen, the Schwann cells were oriented perpendicularly to SMF when they were mixed with collagen,^55^ which indicates that the orientation of cells are strongly affected by their surrounding materials.

**TABLE IV.** Effects of SMFs on nerve cells. Human umbilical vein endothelial cells (HUVECs); oligodendrocyte precursor cells (OPCs); olfactory ensheathing cells (OECs); neural progenitor cells (NPCs); and N/A, not available.

Objects		Magnetic flux density	Magnetic field direction	Exposure time	Effects	References
Humans	Satellite cells	80 mT	Upward	21 days	Increased gene expression of DES, MYF5, MYOD1, MYOG, MYH and ACTA1 as well as enhanced IGF1-induced increase of satellite cell proliferation	56
					No effect on myogenic maturation	57
	Neuronal cells FNC-B4		N/A	120 min	Promoted neural differentiation	52
	PC12, HUVECs	200 mT	Parallel	7 days	Combined MNPs promoted the proliferation and secretion of neurotrophic factors	58
	OPCs	300 mT	Perpendicular	2 h/day, 2 weeks	Enhanced differentiation, promoted function and neurotrophic factor release	53
Rats	Whole animal	10 mT	N/A	3 weeks	Enhanced the generation of new doublecortin-expressing cells in the SVZ and neocortex	49
	OECs	30, 50, and 70 mT	Parallel or perpendicular	36 h	Influenced the division, movement, migration direction and speed of OEC, as well as the formation and rearrangement of microtubules and actin filaments for tissue regeneration	50
	NPCs	100 mT	N/A	12 days	Promoted cellular self-renewal and differentiation into neurons	51
	Schwann cells	8 T	Parallel	60 h	Induced parallel orientation of the Schwann cells along the SMF direction	55
Mice	NPCs	500 mT	N/A	7 days	Affected normal neurogenesis and promoted neurospectral differentiation as well as neuronal maturation	54

Besides using SMFs alone, several studies have combined SMFs with other treatments for neural differentiation and regeneration. For example, Birk *et al.* found that 80 mT SMF stimulated insulin-like growth factor 1 (IGF1)-induced human satellite cell proliferation during the first days of myogenesis.^56^ They also investigated the effects of combining pro-myogenic differentiation hepatocyte growth factor (HGF) at 80 mT SMF on human satellite cell cultures and reported that HGF or HGF + SMF stimulated human satellite cell cultures did not promote myogenic maturation of human satellite cell cultures.^57^ In a recent work, Yang *et al.* performed both *in vitro* and *in vivo* experiments to show that moderate SMFs in combination with magnetic-responsive aligned fibrin hydrogel (AFG) (consists of MNPs uniformly embedded in AFG) can promote nerve regeneration.^58^

## V. MYOBLASTS

There are also some studies reported the effects of SMF on myoblasts, and most of them showed positive effects. For example, Surma *et al.* treated satellite cells with 60–400 *μ*T of weak SMFs and found that the weak SMFs accelerated the development of skeletal muscle cells and led to multinucleated hypertrophic myotube formation.^59^ Bekhite *et al.* found that 1 mT SMF enhanced cardiomyogenesis in Flk-1+ cardiac progenitor cells.^60^ Stern-Straeter *et al.* observed an increase in myotube formation in human satellite cells in ∼80 mT SMF-treated growth medium (GM).^62^ Similarly, Coletti *et al.* found that 80 mT SMF treatment for 5 days accelerated myogenic differentiation of rat L6 myogenic cells and counteracted the inhibition effects of tumor necrosis factor (TNF) on myogenesis.^63^ However, there are also a few negative results. For example, Li *et al.* found that the proliferation, migration, and adhesion potential of human umbilical artery smooth muscle cells (hUASMCs) were significantly decreased after exposure to 5 mT SMF for 48 h.^61^ Kim *et al.* found that 200 mT SMF decreased the growth of cultured myoblast C2C12 cells.^64^

## VI. DENTAL PULP STEM CELLS

There are a few studies about the potential application of SMFs in dental regeneration, especially the effects of SMFs on DPSCs. For example, Na *et al.* found that 1 mT SMF promoted DPSC proliferation, migration, adult dentin differentiation, and mineralization.^65^ Zheng *et al.* reported that 1, 2, and 4 mT SMFs rearranged the actin filaments of DPSCs, effectively induced DPSCs odontogenesis, stimulated the migration, and proliferation of DPSCs.^7^ For stronger magnets, Lew *et al.* also found that 400 mT SMF can enhance DPSC proliferation.^66^ Moreover, they found that 400 mT treatment for 20 days promoted migration and reparative dentin formation.^67^ In addition to DPSCs, Hsu and Chang found that although 290 mT SMF treatment of dental pulp cells (DPCs) for 9 days did not affect their proliferation, the osteogenic differentiation and mineralization were accelerated.^68^

## VII. OTHER STEM CELLS

In addition to bone regeneration, neural differentiation, and wound healing, stem cells research related to SMFs also includes effects on other stem cells (Table V), among which are MSCs. MSCs have the ability to differentiate to multiple cell types and can differentiate into a variety of tissues, such as nerve, heart, bone, and tendon. This ability of multidirectional differentiation provides an important potential for the treatment of many human diseases. For example, Jouni *et al.* found that after culturing rat BMSCs in 4 mT SMF for 96 h, SMF exaggerated the differentiation potential of BMSCs to primordial germ cells (PGCs).^69^ They treated rat BMSCs with 4, 7, and 15 mT SMFs, and found that the cell survival and proliferation were negatively correlated with SMF intensity and duration.^70^ Sarvestani *et al.* reported that 15 mT SMF did not have any detectable impact on the cell cycle of rat BMSCs.^71^ Sadri *et al.* revealed that 18 mT or 24 mT SMFs affected the cell cycle progression, proliferation rate and arrangement of human MSCs, and induced cell differentiation.^72^ Wu *et al.* found that ∼140 mT of SMFs exposure promoted the proliferation of MSCs.^73^ Maredziak *et al.* found that 500 mT SMF treatment for 7 days enhanced the viability and proliferation rate of human ASCs.^6^ Wang *et al.* discovered that after exposing ASCs to 500 mT SMF for 7 days, the cell viability and proliferation were mildly reduced.^74^ They also treated adipose-derived mesenchymal stem cells (AdMSCs) of canine and equine with 500 mT SMF and showed that canine AdMSCs significantly reduced proliferation rate, whereas the proliferation activity of equine AdMSCs was enhanced.^75^ Schäfer *et al.* found that viability, proliferation rate, and the chondrogenic differentiation capacity of superparamagnetic particles of iron oxide (SPIO)-labeled or unlabeled human MSCs were not affected by 600 mT SMF.^76^ Dikina *et al.* found that both static and time-varying magnetic fields generated by 1.44–1.45 T permanent magnets had no effect on cartilage development in human MSCs.^77^

**TABLE V.** Effects of SMFs on stem cells or tissues. Dental pulp stem cells (DPSCs); human umbilical artery smooth muscle cells (hUASMCs); mesenchymal stem cells (MSCs); adipose-derived stem cells (ASCs); bone marrow mesenchymal stem cells (BMSCs); dental pulp cells (DPCs); adipose-derived mesenchymal stem cells (AdMSCs); Schmidtea mediterranea (CIW4); and N/A, not available.

Objects		Magnetic flux density	Magnetic field direction	Exposure time	Effects on cell proliferation and differentiation	Effects	References
Humans	DPSCs	1 mT	N/A	24 h	Promotion	Induction of MAPK pathway-regulated proliferation, migration, osteogenic/dental differentiation and mineralization of DPSCs	65
		1, 2, and 4 mT				Rearranged the actin filaments, effectively induced DPSCs odontogenesis	7
	hUASMCs	5 mT		48 h	Inhibition	Decreased the proliferation, migration, and adhesion potential	61
	MSCs	24 mT	Parallel	24, 36, 48, 60, and 72 h	Promotion	Influenced on alignment and proliferation rate and induction of mRNA expression of Sox-2, Nanong and Oct-4 genes	72
	Adult skin fibroblasts	35–120 mT	Upward and downward	14 days	Inhibition	Reduced the initial attachment and subsequent growth	79
	WI-38						
	Satellite cells	80 mT	Upward	21 days	Promotion	Increased myotube formation	62
	MG63 osteoblast-like cells	100, 250, and 400 mT		24, 48, and 72 h	Inhibition	Increased ALP activity and extracellular matrix release, inhibited cell proliferation	84
	MSCs	∼140 mT	Perpendicular	6, 12, 24, 48, and 72 h	Promotion	Promoted MSCs proliferation and activates the expression of transcription factors that regulate T-type calcium channels and mediated MSCs proliferation via the MAPK signaling pathway	73
	DPSCs	400 mT	N/A	2 days		Affected the cell membrane of DPSC, activated intracellular calcium ions and increased cell proliferation	66
			Upward	20 days		Activation of p38 MAPK-related pathway enhanced DPSC migration and dentinogenesis	67
	ASCs	500 mT	N/A	7 days		Enhanced the viability and proliferation rate	6
	MSCs	600 mT		24 h	No effect	The migration capacity, viability, proliferation rate and the chondrogenic differentiation capacity were not affected	76
		1.44–1.45 T		24 h/day, 5 days/week, 3 weeks		No enhancement of cartilage formation in cellular slices	77
	CD34+	1.5, 3 T	Horizontal	72 h	Inhibition	GMF exposure did not affect cell proliferation	81
	Lung fibroblasts Hel 299	3 T	N/A	2 h	No effect	Had no effect on clonogenic capacity, proliferation or cell cycle	82
	CD34+	10 T	N/A	4 and 16 h	Promotion	Altered gene expression, enhanced MEP differentiation and/or promoted proliferation of bipotent MEP	83
Rats	Satellite cells	60, 120, 160, and 200 *μ*T		7 days		Accelerated skeletal muscle cell development, led to multinucleated hypertrophic myotubes formation and intracellular calcium ion concentration increase	59
	BMSCs	4 mT		96 h		Exaggerated the differentiation potential of BMSCs to PGCs	69
		4, 7, and 15 mT		24, 48, 72, and 96 h	Inhibition	Cell survival and proliferation rates were reduced, and apoptosis occurred in the cells	70
		15 mT		5 h	No effect	No effect	71
	L6	∼80 mT	Upward	5 days	Promotion	Promoted myogenic differentiation and hypertrophy, and counteracted the effects of TNF on myogenesis	63
	DPCs	290 mT		9 days		Accelerated the osteogenic differentiation and mineralization	68
	ASCs	500 mT	N/A	7 days	Inhibition	Inhibition of ASCs viability, proliferation, cytokine secretion, lipogenesis and osteogenic differentiation without causing DNA damage	74
Mice	Flk-1+	<5 mT		1 h/day, 6 days	Promotion	Enhanced cardiomyogenesis	60
	Whole animal	∼100– 200 mT	Upward and downward	3 weeks	Inhibition	Inhibited DNA synthesis and regeneration in hepatocytes	80
	C2C12	200 mT	Alternating magnetic poles	48 h		Decreased the growth of cultured myoblast C2C12 cells	64
Canines and equines	AdMSCs	500 mT	Upward	24 and 168 h	Promotion	Canine AdMSCs significantly reduced proliferation rate, whereas the proliferation activity of equine AdMSCs was enhanced	75
CIW4		<1 mT	N/A	72 h		Altered stem cell-mediated growth	78

Some groups have also tested SMFs on other stem cells. For example, Van Huizen *et al.* found that weak SMFs altered Schmidtea mediterranea (CIW4) stem cell proliferation and subsequent differentiation, and these effects were related to magnetic field strength.^78^ Sullivan *et al.* applied 35–120 mT SMFs to both fetal lung (WI-38) and adult skin fibroblasts and found that SMFs significantly reduced their initial attachment and subsequent growth.^79^ Song *et al.* found that the upward direction ∼100–200 mT SMFs for 3 weeks could inhibit DNA synthesis and regeneration in hepatocytes, which caused detrimental effects on the lifespan of heavy drinking mice.^80^ Iachininoto *et al.* found that 1.5 or 3 T gradient magnetic fields (GMFs) emitted by magnetic resonance imaging (MRI) exposure of the blood donor CD34+ cells *in vitro* for 72 h did not affect the cell proliferation or clonogenic potential.^81^ Schwenzer *et al.* found that electrostatic fields alone and the turbo spin-echo sequence of 3 T SMF had no effect on clonogenic capacity, proliferation, or cell cycle of eugenic human lung fibroblasts.^82^ Monzen *et al.* found that 16 h of 10 T SMF treatment enhanced differentiation of CD34+ cells that were isolated from human placental and umbilical cord blood to megakaryocyte/erythroid progenitors (MEP) and/or promote proliferation of bipotent MEP.^83^

## VIII. ANALYSIS OF THE DIFFERENTIAL EFFECTS AND MECHANISMS

From the information above, it is clear that SMFs can affect multiple systems in our bodies that are related to regenerative medicine. However, the results are not always consistent, which may be attributed to multiple aspects, such as magnetic field parameters, cell and tissue types, and treatment procedure.^4^ For instance, Shang's team treated osteoblasts MC3T3-E1 cells and osteoclasts Raw264.7 cells with SMFs of 500 nT, 0.2 T, and 16 T and not only found that lower vs higher intensity SMFs generated opposite effects, and osteoblasts and osteoclasts also responded total differently to SMFs.^12,13^ Feng *et al.* found it interesting that while both vertically upward and downward SMFs can promote wound healing, the vertically downward SMF is more effective.^48^ Song *et al.* also found that the downward SMF had a promotion effects on the liver regeneration after high dose of alcohol consumption, but not the upward SMFs, which is due to the opposite direction Lorentz forces exerted on the negatively charged DNA during DNA synthesis.^80^

In addition to magnetic field parameters themselves, there are also multiple other factors that have led to the differential results in the literature. For example, Sullivan *et al.* showed that reactive oxygen species (ROS) levels increased 37% in WI-38 cells exposed to SMFs during the first 18 h after seeding, but no elevation in oxidant levels was observed after a prolonged 5-day exposure. They found that SMF exposure decreased cell attachment by <10% in relatively young cultures, but by >60% in later passage cultures.^79^ Similarly, for the same type of cells (BMSCs) and the same SMF intensity (15 mT), Javani *et al.* observed decreased cell survival and proliferation,^70^ but Sarvestani *et al.* concluded that there was no significant change in the cell cycle.^71^ This is actually not surprising because they used different treatment time (96 h in Javani *et al.*'s study while only 5 h in Sarvestani *et al.*'s study) and different measurement readout (cell survival and proliferation in Javani *et al.*'s study while cell cycle in Sarvestani *et al.*'s study). Moreover, Ogiue-Ikeda *et al.* revealed that the spindle shaped smooth muscle A7r5 cells were not oriented after 60 h of exposure to SMF in low-density culture, whereas those that were oriented by the magnetic field only when the cells were actively proliferating at high cell density,^85^ while the mechanism is unknown. To make things even more complicated, Birk *et al.* showed that the same SMF in combination with different factors can result in completely different results.^56,57^ They found that during the first days of myogenesis, the 80 mT SMF stimulated IGF1-induced human satellite cell proliferation and enhanced gene expression of myogenic maturation markers.^56^ However, when they replaced the IGF1 with HGF, they did not observe any significant changes.^57^

While the bioeffects of SMFs still lack consensus, in general, people are trying to investigate the molecular changes after SMF treatment, aiming to understand the underlying mechanism, but has no conclusion yet. In general, multiple cellular events and signaling pathways were found to be changed after SMF exposure. For example, people think that SMFs may accelerate regeneration of bone, nerve, muscle, etc., by influencing expression of markers related to their regeneration, such as Mash1, Math1, alkaline phosphatase (ALP), osteocalcin (OCN), and myogenic factor-5 (MYF5).^14,18,34,51,56,86^ In addition, pathways, such as transforming growth factor-β (TGF-β), bone morphogenic protein (BMP)-Smad, and AKT, are also affected.^7,21,23,24,33,87^ In particular, there are multiple studies that have shown that SMFs influence the proliferation and differentiation of stem cells through MAPK signaling pathway.^36,65–67,69,73^ It is also interesting that receptor activator of NF-κB (RANK), matrix metalloproteinase 9 (MMP9) and V-ATPase, which are related to osteoclasts activation and function, were shown to be upregulated at 500 nT and 0.2 T, but downregulated at 16 T.^12,13^ These opposite changes may contribute to the differential effects of 500 nT, 0.2 T, and 16 T on osteoblast and osteoclast.

In addition to the various reported proteins and pathways mentioned above, SMFs have also been shown to generally affect levels of cellular Ca^2+^ and ROS, which are involved in multiple processes, including cell differentiation and tissue regeneration. For example, multiple studies have indicated that SMFs can affect the stem cell differentiation by altering intracellular Ca^2+^ levels through influencing Ca^2+^ influx and efflux.^59–61,69^ Moreover, SMFs are also indicated to affect tissue regeneration and stem cell differentiation by changing ROS levels.^48,60,78,86,88^ The SMF-induced changes Ca^2+^ and ROS are likely due to the effects of SMFs on cell membrane, electron spin, and radical recombination.^4^ However, as summarized in a previous review,^89^ the effects of SMFs on cellular ROS levels are highly variable, which are dependent on both magnetic field parameters and cell types. In fact, the exact relationship and dose-dependence of SMFs and ROS are still unsolved questions in this field.

However, it should be noted that the above-mentioned molecules and pathways changes are all from the biological point of view. In fact, the most fundamental mechanism lies in physics and biophysics. The mechanisms of SMFs on biological systems mainly include the induction of electric fields and currents, magnetic forces and torques on molecules and cells as well as influence of electron spin states.^78,80,89,90^ For example, SMF can induce the alignment of cytoskeleton due to the magnetic torque and diamagnetic anisotropy.^4^ SMF can modulate ROS level because they can change the electron spin state.^78,80,91^ However, how to fine-tune the SMF parameters to precisely regulate the complicated biological systems is still a challenging question in the field.

## IX. CONCLUSION AND FUTURE PERSPECTIVES

**FIG. 1.**
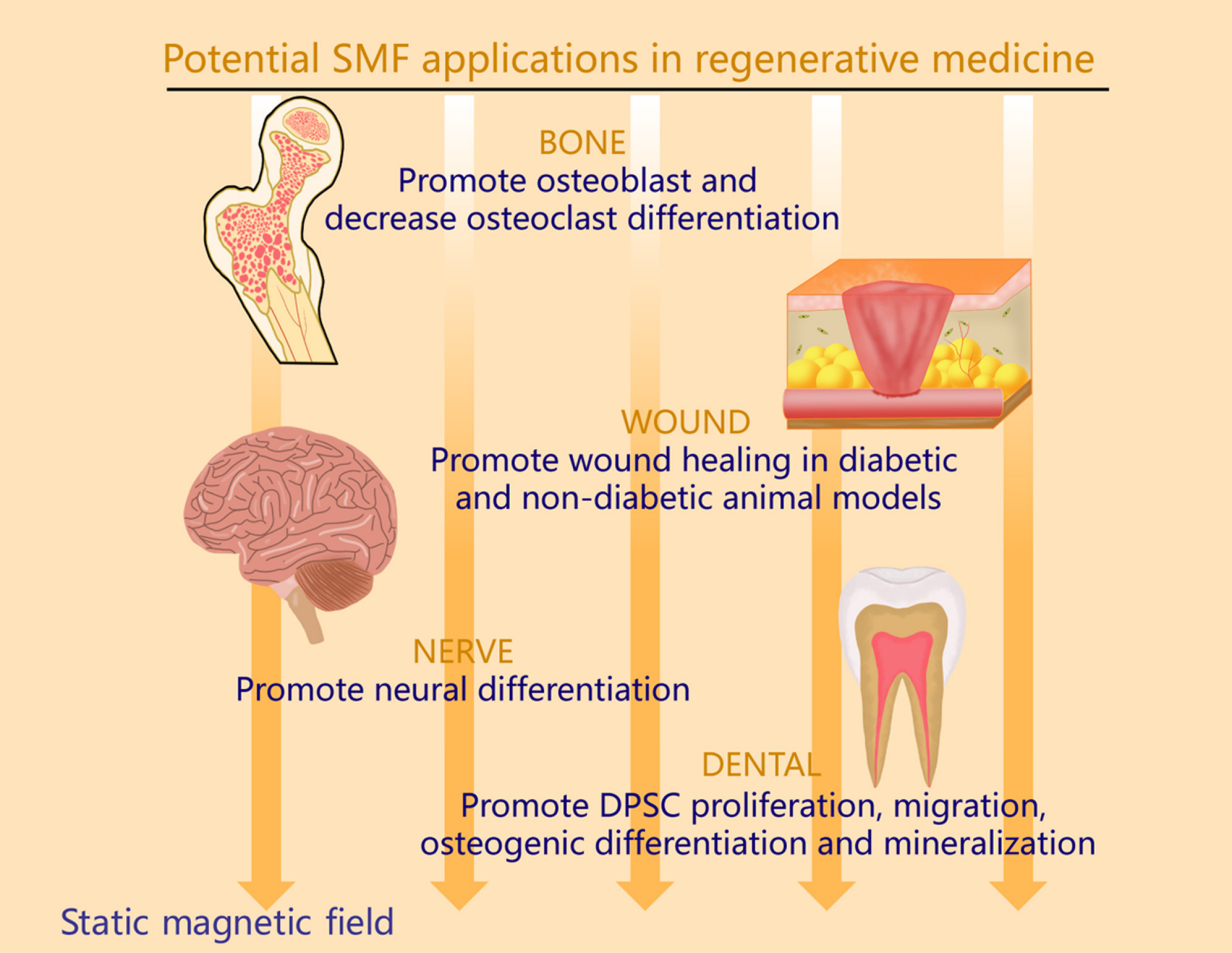
Potential SMF applications in regenerative medicine.

With the advances and increasing demand for regenerative medicine, SMFs have been explored for their potential applications in this aspect. Most current studies were carried out at the stem cell level, and most of which reported positive effects of SMFs on stem cell proliferation, differentiation, and cell survival, while a few studies showed the opposite effects. This variation is likely due to the different SMF parameters, treatment procedures, and cell types, which can all lead to inconsistent outcomes. However, it is interesting that for animal investigations, all studies have shown the beneficial effects of SMFs, especially in prevention/treatment of osteoporosis, fracture healing and bone regeneration, as well as wound healing and dental application (Fig. 1). This is probably due to the fact that animal studies are usually carried out on aspects and parameters that have been proven to be effective in cellular experiments. Based on the reports in the literature, we predict that the applications of 0.1–1 T moderate SMFs on human bodies are likely to produce beneficial effects on bone regeneration and wound healing. From the practical point of view, 0.1–0.5 T SMFs are relatively easy to generate, either by permanent magnets or by electromagnets, which makes them more practical to be used on human bodies. In the meantime, we believe that the potentials of SMFs will be further revealed after more systematic investigations both *in vitro* and *in vivo*, which include identifying systems that can benefit from SMF treatment, optimizing SMF parameters, as well as unraveling the exact physical and biophysical mechanisms that mediated all the reported biological changes. Human studies are strongly encouraged, especially in the fields of osteoporosis, fracture healing, bone regeneration, and wound healing.

## Data Availability

Data sharing is not applicable to this article as no new data were created or analyzed in this study.
